# Vibrationally excited molecule–metal surface reactions in heterogeneous and plasma catalysis: going beyond the Fridman–Macheret *α* model

**DOI:** 10.1039/d5ey00062a

**Published:** 2025-04-22

**Authors:** Nick Gerrits, Annemie Bogaerts

**Affiliations:** a Leiden Institute of Chemistry, Gorlaeus Laboratories, Leiden University P.O. Box 9502 2300 RA Leiden The Netherlands n.gerrits@lic.leidenuniv.nl; b Research Group PLASMANT, Department of Chemistry, University of Antwerp Universiteitsplein 1 BE-2610 Wilrijk Antwerp Belgium; c Electrification Institute, University of Antwerp Olieweg 97 2020 Antwerp Belgium

## Abstract

Vibrational excitation of reactants plays an important role in heterogeneous and plasma catalysis by increasing the reactivity of various rate-controlling steps. Therefore, state-of-the-art microkinetic models attempt to include this effect by modelling the change in reaction rate with the Fridman–Macheret *α* approach. Although this approach is ubiquitous in simulations of plasma catalysis, it is not well established how accurate it is. In this work, we evaluate the Fridman–Macheret *α* approach by comparing it to vibrational efficacies obtained with molecular dynamics simulations. Unfortunately, the agreement is extremely poor (*R*^2^ = −0.35), raising questions about the suitability of using this method in describing vibrationally excited dissociative chemisorption on metal surfaces, as is currently the norm in plasma catalysis. Furthermore, the approach lacks vibrational mode specificity. Instead, we propose an alternative model at comparable computational cost, which is fitted to theoretical vibrational efficacies obtained with molecular dynamics. Our model uses (1) the barrier height to dissociative chemisorption, (2) an indication of how “late” the barrier is, and (3) the overlap of vibrational modes and the reaction coordinate at the barrier. These three features lead to a considerable qualitative and quantitative (*R*^2^ = 0.52) improvement over the Fridman–Macheret *α* approach. Therefore, we advise to make use of our new model, since it can be readily plugged into existing microkinetic models for heterogeneous and plasma catalysis.

## Abstract 2

Vibrational excitation of reactants can play an important role in increasing the reactivity in heterogeneous and plasma catalysis. Here, a critical look is taken on how to model this chemical process.

## Graphical abstract

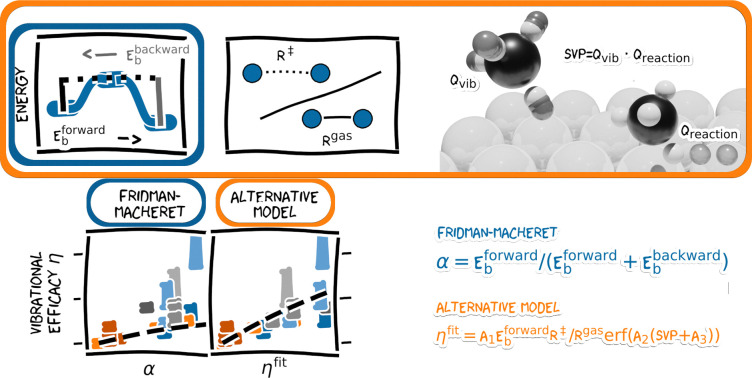

**Broader context**

Plasma catalysis has the potential to convert (green) electricity into valuable chemical products with high efficiency. Unfortunately, for this technology to become mature, a considerable amount of research is still required. For example, the ionized plasma can induce many different changes in both the reactants and the catalyst. One such effect is the vibrational excitation of reactants, which boosts reactivity in heterogeneous and plasma catalysis by mainly affecting key rate-determining steps. To account for this, current microkinetic models often use the Fridman–Macheret *α* approach. While common in plasma catalysis simulations, its accuracy is unclear. In this study, we compare the Fridman–Macheret *α* method with vibrational efficacies from literature molecular dynamics simulations. The results show poor agreement, casting doubt on the method's suitability for describing vibrationally excited reactions on metal surfaces. We propose an alternative model that, at a similar computational cost but with considerable improvements, uses three physical fingerprints of the reaction: (1) the dissociative chemisorption barrier height, (2) the barrier's “lateness”, and (3) the overlap of vibrational modes with the reaction coordinate at the barrier. We recommend adopting this new model, as it can be easily used in existing microkinetic models for heterogeneous and plasma catalysis.

## 1 Introduction: role of vibrational excitation in heterogeneous and plasma catalysis

Dissociative chemisorption (DC) is a chemical process where a reactant (typically a molecule) adsorbs on a surface (often metallic) and simultaneously breaks a bond in the reactant. This is an extremely important reaction in heterogeneous and plasma catalysis, since it is often the rate-controlling step. Therefore, fundamental understanding and predictions of the DC of molecules on metal surfaces is important in order to improve catalysis. For example, how energy is partitioned can affect how chemical reactions proceed. One such important aspect of energy partitioning in catalysis is the vibrational excitation of gaseous reactants, which has been shown to promote the DC of many different molecules on metal surfaces.^1–8^ This effect can especially be important in plasma catalysis, where vibrational excitation is ubiquitous.^9–17^ Likewise, if the rate-controlling step is highly activated and the chemical process is operated under high temperatures, it is likely that vibrational excitation is important in conventional thermal heterogeneous catalysis as well.

**Fig. 1:**
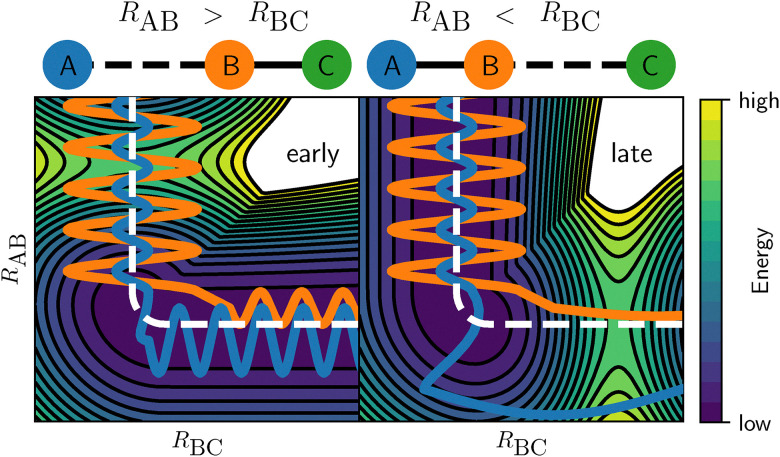
Example of a 2D PES for an early (left) and a late (right) barrier for the reaction of an atom with a diatomic molecule (A + BC → AB + C). The two DOFs shown involve the bond lengths between atoms A and B (*R*_AB_), and atoms B and C (*R*_BC_), *i.e.*, the impact radius and angle of the full 5D system, which are typically employed in gas phase simulations, are not considered. The blue and orange lines exemplify a reactive trajectory of the vibrational ground and excited state, respectively. The colors indicate the relative energy, where the contours are indicated by the black lines, and the MEP is shown as a white dashed line.

Generally, when translational kinetic energy is not so effective in promoting reactivity, vibrational energy is considerably more effective at increasing reactivity than translational energy. This is especially the case for reactions classified as a late barrier system combined with a large barrier height, often referred to as Polanyi's rules.^18^ The reason is that, in such a case, the molecule requires a large amount of energy to overcome the barrier to DC. The minimum energy path (MEP) will then also typically exhibit a large amount of curvature, prior to reaching the transition state (TS). Thus, if the energy in the reactant originates primarily from the translational degrees of freedom (DOFs), it is difficult for the molecule to stick to the MEP and it will “go off the rails”. This deviation from the MEP results in even larger barrier heights that need to be overcome, which is typically referred to as the bobsled effect.^19,20^ An example of this is shown in Fig. 1 (blue line, right panel) for an atom reacting with a diatomic molecule, *i.e.*, A + BC → AB + C. If instead a large portion of the energy comes from the vibration, it is easier to round the corner and the molecule will manage to remain closer to the MEP, resulting in lower accessible barrier heights (Fig. 1, orange line, right panel). Of course, for an early barrier system, the reverse holds true (Fig. 1, left panel): vibrational energy is not effective in promoting reactivity, because its motion is perpendicular to the reaction coordinate at the TS and parallel with the dividing surface that defines the barrier. In other words, the vibrational energy cannot be used to overcome the barrier. Translational energy, on the other hand, is now perpendicular to the barrier and thus more effective at promoting reactivity. Additionally, there is no “corner” in the MEP prior to the TS, ensuring that the bobsled effect does not affect the reactivity. For the DC of a diatomic molecule, Fig. 1 remains similar, but atom A and the bond length *R*_AB_ are typically replaced by the surface and the distance between surface and molecule BC, respectively. Similar potential energy surface (PES) cuts can be made for polyatomic molecules in reduced dimensionality, by only varying the distance between the molecule and the surface as well as the dissociating bond length, while all other DOFs are either relaxed or fixed at their TS value.

## 2 Microkinetic models in plasma catalysis and the effect of vibrational excitation

Microkinetic models (MKMs) have been invaluable in understanding the reactivity of metal catalysts under realistic catalytic conditions. An MKM simulates a chemical process as a network of elementary reaction steps using rate equations. This way, the effect of catalytic conditions like concentration, temperature, and pressure on the entire chemical process can be investigated, but also the effect of changing the catalyst itself. Moreover, it can provide insights on the reaction pathways. Often, the Arrhenius equation is employed to obtain the rate coefficient *k* for an elementary reaction step:1
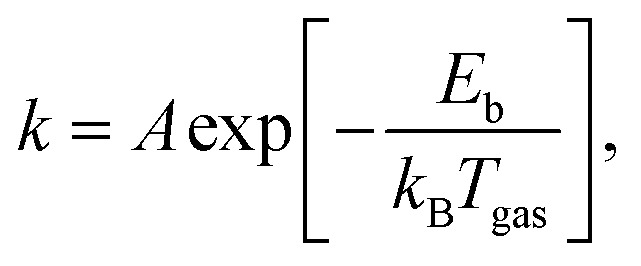
where *A* is the pre-exponential or frequency factor, *E*_b_ the barrier height, *T*_gas_ the gas temperature, and *k*_B_ the Boltzmann constant. The exponent computes the fraction of reactants that have enough energy, assuming thermal equilibrium, to overcome the barrier. Of course, many corrections can be made to eqn (1) to account for a myriad of effects, *e.g.*, lateral adsorbate interactions,^21,22^ recrossing dynamics,^23^ and tunneling.^24–26^

## 3 Evaluation of the Fridman–Macheret *α* model

As discussed above, it can be important to capture the effect of vibrational excitation in MKMs, which is typically approximated with the Fridman–Macheret (FM) *α* approach.^27,28^ This approach was originally designed for the gas phase reaction A + BC → AB + C depicted in Fig. 1 and tries to enforce Polanyi's rules, by assuming that the “lateness” of the barrier determines the effectiveness of vibrational energy relative to translational energy. Furthermore, it is assumed that the lateness of the barrier and the ratio between the forward and backward barrier heights are proportional. By computing this ratio, the so-called vibrational yield *α* is obtained:^27^2
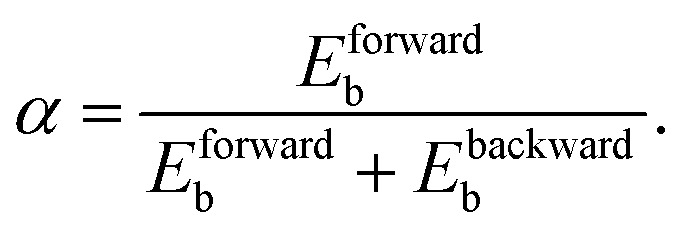


Subsequently, eqn (1) is modified to reduce the effective barrier height by the vibrational energy *E*_*ν*_ as follows:^28^3
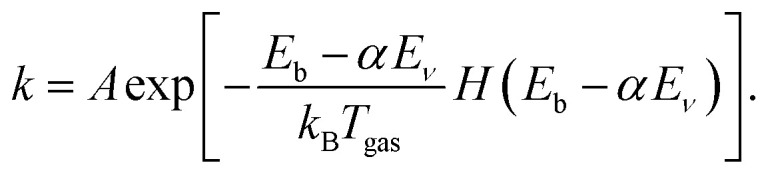
*H* is the Heaviside function to ensure that the exponent is bounded by unity. It should be noted that this might not reflect reality in the sense that the barrier height might not actually change considerably (*e.g.*, if the bobsled effect is small), but that more energy can be converted into the reaction coordinate. Nevertheless, the effect is the same: an increase in the reaction rate is obtained, in accordance with experimental and theoretical studies.

Mehta *et al.* were the first to apply the FM *α* approach to molecule–metal surface reactions, instead of gas phase reactions.^28^ By doing so, they predicted that plasma catalysis can break away from the traditional scaling relations present in thermal heterogeneous catalysis, which was also supported by experiments.^14,28^ This work fueled many subsequent theoretical studies that employed the FM *α* approach, providing valuable new insights into plasma catalysis.^17^ For example, Ma *et al.* were able to rationalize their experiments on the plasma-catalytic oxidation of N_2_ with such simulations.^29^ For the plasma-catalytic synthesis of ammonia, Engelmann *et al.* modelled both vibrational excitation and radical-induced Langmuir–Rideal reactions, where a gas phase reactant reacts directly with an adsorbate, *i.e.*, without thermalization or adsorption prior to the reaction.^30^ (We note in passing that the Langmuir–Rideal reaction mechanism, also an important reaction process in plasma catalysis, is often erroneously referred to as the Eley–Rideal mechanism.^31,32^ However, Eley worked on a different mechanism, namely the reaction between a physisorbed and a chemisorbed adsorbate that are in thermal equilibrium with the surface.^32,33^) Their conclusion was that radical reactions were generally much more important than vibrational excitation. Similarly, for CO_2_ hydrogenation, Michiels *et al.* concluded that plasma-generated radicals and reaction intermediates were orders of magnitude more effective at increasing the turnover frequency than vibrational excitation.^34^ In contrast, for the non-oxidative coupling of methane, vibrational excitation was found to be considerably more competitive with other reaction channels, depending on the binding strength of the catalyst surface.^35^ Furthermore, the FM *α* approach allowed Engelmann *et al.* to make predictions for the optimal plasma conditions.^35^ Although the overall effect was again limited, Loenders *et al.* predicted that vibrational excitation could improve the selectivity of certain products in the partial oxidation of methane on Pt(111).^36^ In short, the FM *α* approach has enabled theoretical studies of vibrational excitation in plasma catalysis. It should also be noted that this approach has only been applied to vibrational excitation of gaseous reactants, and not to adsorbates. Vibrational excitation and subsequent reaction of adsorbates would involve very different reaction dynamics and likely require different models than those discussed in this work.^37–39^

It is clear that the Fm *α* approach is a computationally attractive model for plasma catalysis. Unfortunately, the model has several fundamental shortcomings, which we will discuss now. The determination of the vibrational yield *α* relies only on the ratio between the ground state forward and backward barrier heights. It is assumed that the position of the TS along the reaction coordinate is related to this ratio, but unfortunately there is no guarantee that this assumption is correct. Furthermore, there is no dependence on the actual vibrational mode other than the excitation energy *E*_*ν*_. Unfortunately, depending on the specific vibrational mode(s) being excited, vibrational excitation of polyatomic molecules often leads to very different reactivity. For example, for the DC of methane on several metal surfaces, excitation of the stretch modes is considerably more effective than the bend modes.^1,40–44^ Conversely, for CO_2_ + W(110), the bending mode has been found to be more effective.^45^ Moreover, the reaction of vibrationally excited methane can become more sterically hindered, since the excitation can be comparatively local, *i.e.*, the four CH bonds are not necessarily energized in the same way.^46^ Similarly, overtone excitation leads to complex vibrational distributions, where the localization can occur in a more localized or delocalized manner, again affecting reactivity of the CH bonds differently.^44^ The effect of vibrational excitation is also dependent on the specific vibrational level, because overtones do not necessarily yield, at least quantitatively, the same effect.^4^ Additionally, since *α* ∈ [0,1], vibrational energy can never be more effective than translational energy. Yet, many instances have been discovered where vibrational energy is actually more effective.^7^ Again for methane, it is often observed that excitation of the stretch mode is more effective than increasing the translational energy.^3–5,47,48^ Likewise, for the excitation and dissociation of the CO bond in methanol on Cu(111), vibrational energy was found to be remarkably 3.4–5.3 times as effective as translational energy at promoting the reaction.^49^

**Fig. 2:**
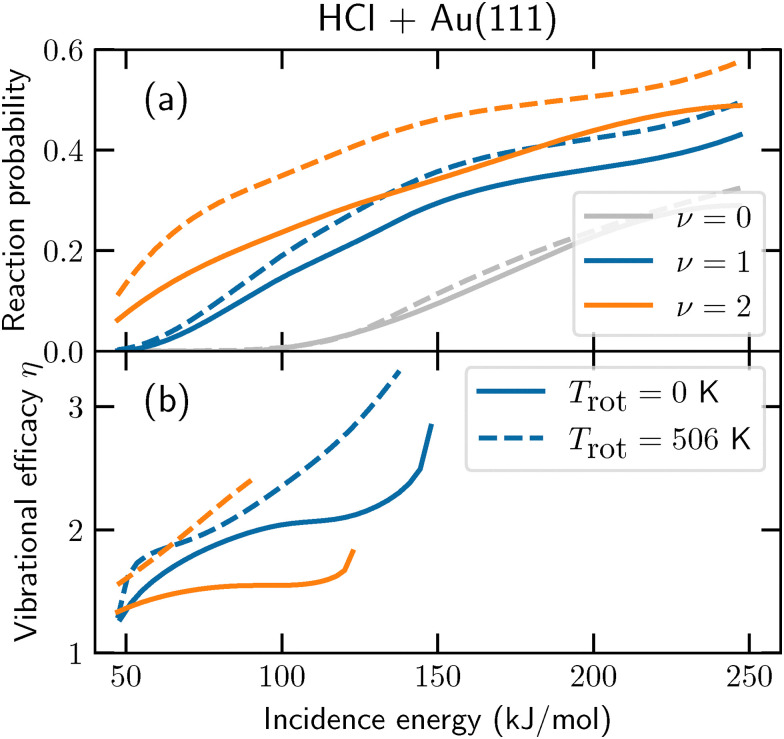
(a) Reaction probabilities for normally incident HCl on Au(111) and (b) the corresponding vibrational efficacies (*η*). Results for *ν* = 0, 1, and 2 are shown in grey, blue, and orange, respectively. The vibrational efficacy is computed relative to *ν* = 0 with the same rotational state Boltzmann distribution. The solid (dashed) lines indicate results for *T*_rot_ = 0 K (506 K). This figure has been adapted from ref. 6

Another issue with the Fm *α* approach is that it assumes that the effect of vibrational energy is independent of other DOFs. However, for HCl + Au(111), a large synergistic effect between vibrational and rotational excitation is observed (see Fig. 2).^6^ From the initial rovibrational state-selected reaction probability *R*_*ν*,*J*_(*E*_i_), the so-called vibrational efficacy *η* (which measures how effective increasing the vibrational energy is at promoting the reaction relative to increasing *E*_i_ for the reactant in the state *ν*,*J* to obtain the reaction probability *R*) is determined as follows:4
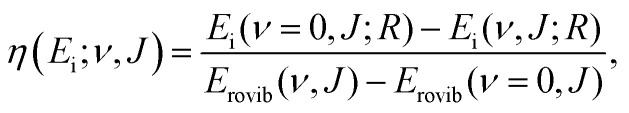
where *E*_i_(*ν*,*J*;*R*) is the incidence energy at which *R*_*ν*,*J*_(*E*_i_) = *R*, and it is assumed that *R*_*ν*,*J*_(*E*_i_) is a bijective or invertible function, *i.e.*, only one value of *E*_i_ corresponds to a particular value of *R*_*ν*,*J*_. The vibrational efficacy *η* can be used to replace *α* in eqn (3) to achieve a similar effect, *i.e.*, describing the increase in reaction rate due to vibrational excitation. By increasing the rotational temperature, it is observed that the vibrational efficacy increases considerably as well (Fig. 2(b)), even though the amount of added rotational energy is comparatively small, showing an important relation between the vibrational and rotational DOFs. Similarly, the effectiveness of vibrational energy in HCl is also dependent on the translational energy, which is observed for other molecule–metal surface reactions as well. In short, the FM *α* approach neglects many important fundamental aspects of how vibrational energy can influence molecule–metal surface reaction rates.

**Fig. 3:**
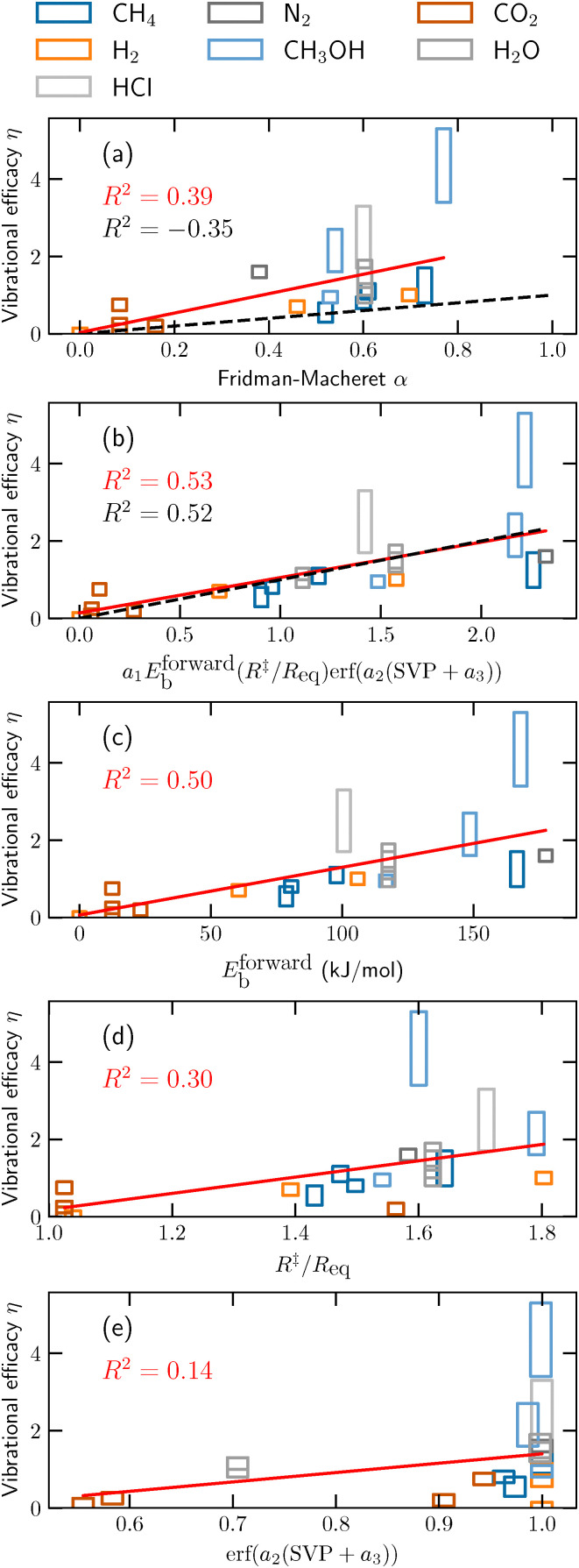
Vibrational efficacies (*η*, rectangles) obtained from MD studies of the DC of various molecules on metal surfaces (see Table 1 for the data and their references). The black dashed lines are the efficacies as predicted by the FM *α* model (panel a) and our alternative approach (panel b), whereas the red solid lines are linear regression fits to the vibrational efficacies. The (color coded) *R*^2^ values are indicated in the figure. Panels c–e show the correlation between *η* and the three individual parts of our alternative approach (forward barrier height, ratio between dissociating bond lengths in the gas phase and TS, and SVP of the vibrational mode, respectively). In other words, the linear regression fits in panels c–e employ different weights compared to the fit to the full model in panel b.

As discussed above, vibrational efficacies (*η*) can be extracted from molecular dynamics (MD) simulations with eqn (4). In Fig. 3(a) and Table 1, we compare the FM *α* model with the extracted efficacies *η*, as found in literature. If *η* is missing from literature, we compute it from published vibrational state-resolved reaction probabilities, by computing the range of energy shifts between the published continuous curves or discrete data points. The *R*^2^ value between the two is only −0.35 (see the black dashed line compared to the rectangles in Fig. 3(a)), signaling an extremely poor agreement. Overall, the FM *α* approach underestimates vibrational efficacies considerably. Even if *α* is reweighted through a linear regression, an *R*^2^ of only 0.39 is obtained (see red solid line). We expect that approaches that attempt a similar reweighting (as suggested by, *e.g.*, Kunova *et al.*^50^) would achieve similar limited success. Furthermore, the FM *α* approach does not consider vibrational mode specificity. It is clear that the need for a way to include the effect of vibrational excitation on reaction rates is large, but that the workhorse FM *α* approach is unsuitable for, at least, DC. Therefore, we now turn to potential alternatives.

**Table 1:** Literature values for the FM *α*, vibrational efficacy *η*, *η*_fit_ predicted by eqn (5), forward barrier height *E*^forward^_b_ (kJ mol^−1^), ratio between the dissociating bond length at the TS and gas phase equilibrium geometry *R*^‡^/*R*_eq_, and SVP value for several vibrational state-resolved molecule–metal surface reactions. If *η* is not reported in the reference, we compute it ourselves from the range of energy shifts between the reported vibrational state-specific reaction probability curves or data points (depending on what is available) in the reference, using eqn (4). Due to a general lack of published SVP data, the values for H_2_ are taken from H_2_ + Ag(111),^51^ CH_4_ + Ni(211) is taken from CH_4_ + Pt(111)/Ni(111),^52^ and CHD_3_ + Cu(111) is estimated from the elbow plot of the TS in ref. 53 and CHD_3_ + Ni(111).^54^ For N_2_ + Ru(0001) and HCl + Au(111), we have computed the SVP values with the computational setup of ref. 55 and 56, respectively

Molecule (excitation) + metal surface	FM *α*	*η*	*η* ^fit^	*E* ^forward^_b_	*R* ^‡^/*R*_eq_	SVP
CHD_3_ (s. stretch) + Pt(111)	0.52^3^	0.3–0.8^57^	0.91	78.7^3^	1.43^3^	0.44^42^
CHD_3_ (s. stretch) + Ni(111)	0.61^58^	0.9–1.3^57^	1.19	97.9^48^	1.47^48^	0.84^54^
CH_4_ (s. stretch) + Ni(211)	0.60^59^	0.8–1.0^59^	0.96	80.6^59^	1.50^59^	0.40^52^
CHD_3_ (s. stretch) + Cu(111)	0.73^53^	0.8–1.7^4^	2.26	166.6^53^	1.64^53^	0.90^53,54^
H_2_ + Al(110)	0.70^60^	1.0^60^	1.58	105.9^60^	1.80^60^	0.90^51^
H_2_ + Cu(111)	0.46^61^	0.7^62^	0.70	60.6^63^	1.39^63^	0.90^51^
H_2_ + Pt(111)	0.00^64^	0.0–0.2^65^	0.00	0.0^64^	1.04^64^	0.90^51^
HCl + Au(111)	0.60^66^	1.7–3.3^6^	1.42	100.6^56^	1.71^56^	0.94
N_2_ + Ru(0001)	0.38^67^	1.6^68^	2.32	177.5^63^	1.58^63^	0.94
CH_3_OH (CO stretch) + Cu(111)	0.77^49^	3.4–5.3^49^	2.22	167.9^49^	1.60^49^	0.75^49^
CH_3_OH (OH stretch) + Cu(111)	0.54^49^	1.6–2.7^49^	2.17	148.6^49^	1.79^49^	0.51^49^
CH_3_OH (CH stretch) + Cu(111)	0.53^49^	0.8–1.1^49^	1.48	116.7^49^	1.54^49^	0.87^49^
CO_2_ (bend) + Ni(100)	0.16^69^	0.2–0.4^69^	0.27	23.2^69^	1.56^70^	0.28^70^
CO_2_ (as. stretch) + W(110)	0.08^45^	0.1–0.3^45^	0.06	12.5^45^	1.03^45^	0.02^45^
CO_2_ (s. stretch) + W(110)	0.08^45^	0.1–0.4^45^	0.06	12.5^45^	1.03^45^	0.03^45^
CO_2_ (bend) + W(110)	0.08^45^	0.6–0.9^45^	0.10	12.5^45^	1.03^45^	0.35^45^
H_2_O (stretch) + Cu(111)	0.60^71^	1.3–1.9^72^	1.57	117.6^72^	1.62^71^	0.70^71^
H_2_O (bend) + Cu(111)	0.60^71^	1.0–1.3^72^	1.11	117.6^72^	1.62^71^	0.10^71^
D_2_O (stretch) + Cu(111)	0.60^71^	1.2–1.7^73^	1.57	117.6^72^	1.62^71^	0.70^71^
D_2_O (bend) + Cu(111)	0.60^71^	0.8–1.3^73^	1.11	117.6^72^	1.62^71^	0.10^71^

## 4 Our alternative approach

In principle, the most obvious way to compute accurate reaction rates is to perform vibrational state-specific MD simulations.^7^ These dynamical calculations do not require approximations to the reaction rate equation, because the obtained reaction probabilities can be directly converted into reaction rates.^74^ However, such simulations are not trivial to perform, nor are they typically computationally tractable (even with machine learning), especially for a complete reaction network. As such, for now, more approximate methods are required to improve the predictions made by MKMs.

In this work, we propose to use a combination of (1) the forward barrier height (*E*^forward^_b_), (2) the ratio of the dissociating bond length between the TS and the reactant (*R*^‡^/*R*_gas_), and (3) the sudden vector projection (SVP) approach, to obtain vibrational state-specific efficacies for use in MKMs. These three “features” can be determined with comparatively standard density functional theory (DFT) calculations (similar as used in determining the FM *α*), which we will discuss in detail further below. This combination has been fitted to the average vibrational efficacies *η* reported in Table 1 as follows:

5 *η*^fit^ = *a*_1_*E*^forward^_b_(*R*^‡^/*R*_eq_)erf(*a*_2_(SVP + *a*_3_)),

where erf is the error function, yielding *a*_1_ = 0.008259 mol kJ^−1^, *a*_2_ = 2.4405, and *a*_3_ = 0.2032. In Fig. 3(b) and Table 1, we see that the agreement between our model and the vibrational efficacies obtained with MD (*R*^2^ = 0.52) is considerably improved over the FM *α* model. Moreover, we emphasize that our proposed model includes dependence on the shape of the PES and concomitant geometry of the TS as well as the overlap of vibrational modes, making it system and vibrational mode specific and thus much more suitable for polyatomic molecules. Considering both the qualitative and quantitative improvements that our model brings, we propose to use *η*^fit^ determined through eqn (5) of the FM *α*. MKMs that employ eqn (3) to describe vibrational excitation-mediated reactions, which can be important in, *e.g.*, plasma catalysis, should yield improved results. Because the FM *α* and our proposed *η*^fit^ require in principle the same DFT calculations, the computational cost remains essentially the same.

### 4.1 Explanation of the individual features of our new model

We examine the three individual features of eqn (5) in Fig. 3(c)–(e), by fitting a linear regression to the individual features *E*^forward^_b_, (*R*^‡^/*R*_eq_), and *a*_2_erf(SVP + *a*_3_), *i.e.*, the linear regression fits deviate from the fit in Fig. 3(b) (red line). The fitted slope *a* and intercept *b* of the linear regression fits are *a* = 0.012335 mol kJ^−1^ and *b* = 0.0647 (panel c), *a* = 2.109 and *b* = −1.929 (panel d), and *a* = 2.423 and *b* = −1.023 (panel e), compared to *a* = 0.914716 mol kJ^−1^ and *b* = 0.140 in panel b. The first feature of our model is the forward barrier height *E*^forward^_b_. It should be noted that this barrier height is also employed in the FM *α* approach, but only to approximate the “lateness” of the TS. However, in Fig. 3(c), we can observe that the vibrational efficacy correlates considerably more with the absolute forward barrier height (*R*^2^ = 0.50) than the ratio between the forward and backward barrier heights as employed in the FM *α* model (*R*^2^ = −0.35). In general, the larger *E*^forward^_b_ is, the larger *η* is. Moreover, the second feature employs geometric details of the reactant and the TS, by computing the ratio of the dissociating bond length between the reactant and the TS (*i.e.*, *R*^‡^/*R*_eq_) to estimate the lateness of the barrier. This seems much more effective (*R*^2^ = 0.30, Fig. 3(d)) than employing the ratio between the forward and backward barrier heights.

The final feature we include in our model is aimed at incorporating vibrational mode specificity. The SVP model^75^ might be a good starting point for obtaining reasonable predictions on vibrational mode specific excitation at a limited computational cost. In this model, the overlap between the gas phase reactant's vibrational modes and the direction of the reaction coordinate at the TS (*i.e.*, the imaginary mode) are computed, with the requirement that the reaction occurs rapidly and without equilibration. For DC, this requirement is often fulfilled, although exceptions are known. For example, the DC of ammonia on Ru(0001) is rapid and direct (*i.e.*, not trapping mediated), but it proceeds through a precursor state before it reaches the TS. It is suspected that this precursor state scrambles the vibrational energy, which leads to vibrational efficacies always being unity.^76,77^ Thus, we have excluded such reactions from the data our model is fitted to, focusing on direct, rapid DC without precursor states. In the SVP approach, vibrational modes that yield a small overlap with the reaction coordinate are expected to contribute little to the reactivity, as has indeed been observed in MD simulations (see, *e.g.*, Table 1). Opposite to the FM *α* model, this allows for vibrational mode-specific predictions. Moreover, the SVP model has an explicit dependence on the shape of the PES at the TS, which is also lacking in the FM *α* model. Even though the correlation between the vibrational efficacies and erf(*a*_2_(SVP + *a*_3_)) is still very poor (*R*^2^ = 0.14, see Fig. 3(e)), it does seem to provide improvement over the FM *α* approach (*R*^2^ = −0.35) when combined with *E*^forward^_b_ and *R*^‡^/*R*_eq_ (*R*^2^ = 0.52). Without reweighting or refitting, the SVP approach yields values in the interval [0,1], because it is a dot product between the unit vectors of two vibrational modes. However, as can be seen in Fig. 3(e) and Table 1, an SVP value close to zero should not necessarily yield a low vibrational efficacy. For example, even though the SVP value for the bending modes of water on Cu(111) is low (0.10), the vibrational efficacy is large (0.8–1.3). Overall, we find that a shifted sigmoidal function (like the error function erf(*a*_2_(SVP + *a*_3_)) employed here), combined with the other two features, yields a qualitative improvement compared to a linear relationship, without worsening the overall quantitative agreement. Another benefit is that in many cases the computational cost of the SVP approach is negligible, because the necessary vibrational eigenvectors are often already computed to obtain thermodynamic corrections for the reaction network, or at least to confirm whether the TS is a first-order saddle point. Also, since there is a reliance on the computed vibrational eigenvectors, isotopologues can be distinguished as well.

### 4.2 Limitations of our model and outlook

Currently, our model still considerably underestimates the reactivity of HCl + Au(111) and CH_3_OH (CO) + Cu(111) (see Fig. 3 and Table 1). For the former reaction, it is well understood that dynamical effects along the reaction coordinate, prior to the TS, are the cause of the comparatively large vibrational efficacy.^6^ Specifically, the molecule needs to undergo rapid angular reorientation, or it will hit a repulsive wall and scatter back into the gas phase. Both vibrational and rotational motion aid in this reorientation, but in a complex manner that is not captured by the TS and concomitantly our model. For CH_3_OH + Cu(111), we suspect that the stereodynamics prior to the TS of breaking the CO bond are also heavily constrained.^5,49^ Therefore, future models might be improved by somehow including the curvature along and perpendicular to the MEP. If the curvature is decomposed into the relevant energetic DOFs and projected onto the corresponding reactant's inertia, it is likely that this model would retain its vibrational mode specificity and describe dynamical effects better. Subsequently, the model might be able to better distinguish between high and extremely high vibrational efficacies. For example, *η*^fit^ for CHD_3_ + Cu(111) and N_2_ + Ru(0001) (*i.e.*, examples of high vibrational efficacy, see Table 1) is likely to decrease and improve the agreement with MD, because for these systems such dynamical curvature effects are considerably less severe. Possibly, this approach would enable prediction of rotational efficacies as well, allowing for rovibrational state distribution dependent MKMs. We note that similar approaches have been successfully employed in reaction path Hamiltonian simulations of molecule–metal surface reactions, where a harmonic approximation to the expansion of the DOFs along the 1D reaction coordinate is employed.^78,79^

Furthermore, MD studies are often still limited in their accuracy, therefore also limiting the accuracy of our model compared to experiments.^7^ For example, if the charge transfer at the TS is large, the self-interaction error in DFT is also large.^80^ Unfortunately, the workhorse generalized gradient approximation density functionals employed in plasma catalysis are expected to underestimate the barrier height if the charge transfer is large.^81^ Similarly, the employed dynamical approach can sometimes cause issues with zero-point energy conservation and artificial intramolecular vibrational energy redistribution, again affecting the computed reaction probabilities.^82^ Still, the MD simulations tend to yield qualitatively and semi-quantitatively correct results, in reasonable agreement with experiments. As such, future methodological developments in simulations of DC and concomitant improvement of the data should lead to an improvement of our model as well.

Finally, gas phase reactions occurring in, *e.g.*, plasma, atmospheric and astrochemistry often exhibit similar dynamical behaviour as DC in heterogeneous and plasma catalysis.^83^ Therefore, we suspect that if our model is refitted for gas phase reactions, it might be also suitable for MKMs that describe gas phase reactivity.

## 5 Conclusions: modelling of vibrational excitation in heterogeneous and plasma catalysis

In short, simulations and understanding of DC of (ro)vibrationally excited reactants in heterogeneous and plasma catalysis are of fundamental and practical importance. The FM *α* approach is the current workhorse method for simulating the effect of vibrational excitation in MKMs. In this work, we have compared literature values of vibrational efficacies *η*, which describe the change in reactivity due to vibrational excitation, obtained by MD simulations, with the FM *α* approach. Since the agreement is extremely poor (*R*^2^ = −0.35), we conclude that this approach is unsuitable for DC. Fortunately, we are able to provide an alternative model that is based on a few physical fingerprints of the reaction: (1) the forward barrier height, (2) the “lateness” of the TS (akin to Polanyi's rules^18^), and (3) the overlap of the excited vibrational mode with the reaction coordinate at the TS using the SVP approach.^84^ This model yields considerably improved agreement with MD (*R*^2^ = 0.52). Furthermore, opposite to the FM *α* approach, our model is suitable for polyatomic molecules, because it is vibrational mode specific. Considering the similar computational costs of the two approaches, in addition to the qualitative and quantitative improvements, we advice to use our proposed model (*η*^fit^) in MKMs instead of the FM *α* approach.

Unfortunately, the required data to further improve our model for *η*^fit^ is not publicly available and requires considerable work, perhaps in addition to performing more MD studies and improvement of the underlying simulation techniques. The lack of data also precludes application of other (machine learning) data sciences. Nevertheless, our simple model is physically motivated and can be used with comparative ease. Moreover, our proposed future improvements might lead to accurate, computationally cheap reaction rates for a diverse set of reaction steps, which are dependent on both the rotational and vibrational state. This should lead to next-generation MKMs in (plasma) catalysis, which are not only quantitatively more accurate, but can hopefully also lead to new insights. Fitting our model to gas phase data might make it suitable for, *e.g.*, plasma, atmospheric and astrochemistry as well.

## Conflicts of interest

There are no conflicts to declare.

## Data Availability

All data and their source (see, *e.g.*, Table 1) is present in the paper.
